# Long-Term Determinants of the Seroprevalence of the Hepatitis E Virus in Wild Boar (*Sus scrofa*)

**DOI:** 10.3390/ani11061805

**Published:** 2021-06-17

**Authors:** Patricia Barroso, María A. Risalde, Ignacio García-Bocanegra, Pelayo Acevedo, José Ángel Barasona, Javier Caballero-Gómez, Saúl Jiménez-Ruiz, Antonio Rivero-Juárez, Vidal Montoro, Joaquín Vicente

**Affiliations:** 1 Instituto de Investigación en Recursos Cinegéticos (IREC) CSIC-UCLM-JCCM, 13071 Ciudad Real, Spain; pbarrososgg@gmail.com (P.B.); pelayo.acevedo@uclm.es (P.A.); saul.jimenez.ruiz@gmail.com (S.J.-R.); vidal.montoro@uclm.es (V.M.); joaquin.vicente@uclm.es (J.V.); 2 Grupo de Investigación en Sanidad Animal y Zoonosis (GISAZ), Departamento de Anatomía y Anatomía Patológica Comparadas, Facultad de Veterinaria, Universidad de Córdoba, 14014 Córdoba, Spain; 3 Unidad de Enfermedades Infecciosas, Grupo de Virología Clínica y Zoonosis, Instituto Maimónides de Investigación Biomédica de Córdoba (IMIBIC), Hospital Universitario Reina Sofía, 14004 Córdoba, Spain; javiercaballero15@gmail.com (J.C.-G.); arjvet@gmail.com (A.R.-J.); 4 Grupo de Investigación en Sanidad Animal y Zoonosis (GISAZ), Departamento de Sanidad Animal, Universidad de Córdoba, 14014 Córdoba, Spain; v62garbo@uco.es; 5 VISAVET, Animal Health Department, Veterinary School, Complutense University of Madrid, 28040 Madrid, Spain; joseangel.barasona@gmail.com; 6 Escuela Técnica Superior de Ingenieros Agrónomos, UCLM, 13071 Ciudad Real, Spain

**Keywords:** hepatitis E virus, long-term, public health, shared infection, wild boar, zoonosis

## Abstract

**Simple Summary:**

The hepatitis E virus (HEV) is an emerging multi-host pathogen whose main reservoir is suids, and the leading cause of acute viral hepatitis in humans. This study evaluates the main long-term drivers of the exposure to HEV are in the wild boar population from Doñana National Park (southwestern Spain) during a 13-year period (2005–2018). For this purpose, we assay sera from 700 wild boar in which anti-HEV antibodies are widely distributed (46.7 ± 3.8%, 327 out of 700 sampled). The observed marked interannual fluctuations could be explained by the variations in the population control of the wild boar during the study period and its impact on abundance rates. Several factors operating in the medium and long-term (individual, environmental, populational and stochastic) and their interplay explained the exposure to HEV in wild boar. The preferential use of certain areas by wild boar together with its abundance and the meteorological conditions may be behind the level of exposure. Wild boar population control remains a challenge at the international level, and an increase of shared pathogen-related conflicts associated with this species is expected, as exemplified by HEV.

**Abstract:**

The hepatitis E virus (HEV) is an emerging zoonotic pathogen whose main reservoir is suids. Most of the ecological and epidemiological aspects of its sylvatic cycle remain unknown. Thus, in this work, we study the drivers of HEV exposure in the wild boar population of Doñana National Park (DNP, southwest Spain) operating in the medium and long-term (2005–2018). Anti-HEV antibodies are widely distributed throughout the wild boar (46.7 ± 3.8%, 327 out of 700 sampled), showing a statistically significant age-increasing pattern. The temporal pattern displayed important interannual fluctuations. This could be mediated by marked variations in the population control of the wild boar, and subsequent changes in abundance rates, and its interplay with climatic conditions; as wet years together with a low abundance of wild boar led to the lowest seroprevalence. The fact that seroprevalence is high during conditions of high abundance, and not affected by rainfall level, is probably due to the increased interactions among the animals, and possibly, the subsequent higher environmental contamination with HEV particles. The proximity to the marshland (the main water body of the study area) is associated with a higher risk of testing positive, which is probably mediated by the preferential use of this area during the dry season and the favourable environmental conditions for the survival of HEV particles. A deeper understanding of the epidemiology of HEV in host communities deserves future research concerning other susceptible species. Most importantly, wild boar population control remains a challenge at the international level, and an increase of shared pathogen-related conflicts associated with this species is expected, as exemplified by HEV. Therefore, surveillance of wild boar diseases, including integrated population monitoring and sustainable population control programmes, will be essential to control the associated risks.

## 1. Introduction

The hepatitis E virus (HEV, RNA virus, genus Orthohepevirus, family Hepeviridae) [1] is an emerging zoonotic pathogen with a widespread distribution throughout the world [2]. HEV is one of the main causes of acute hepatitis worldwide, affecting more than 20 million people annually [3]. HEV is a multi-host pathogen classified into eight genotypes (HEV-1 to 8), of which only genotypes 1 and 2 affect humans exclusively, while the rest have been isolated in a wide range of wild and domestic animals [4]. Pig (*Sus scrofa domesticus*) and wild boar (*Sus scrofa*) are considered the main animal reservoir species of the virus [5,6], and cases of zoonotic transmission from these species have been detected [7,8].

HEV infection is primarily transmitted via the faecal-oral route through contaminated water or food in epidemic genotypes [1], whereas the transmission routes of zoonotic genotypes also include consuming raw meat or products derived from infected animals, shellfish or vegetables [9]. The shedding of enterically excreted HEV particles into the environment plays a major role in the transmission of the virus, HEV sequences have been detected as clusters in human, swine and wildlife from the same geographical region in sewage, surface and wastewater [10]. Viral particles of HEV have also been found through molecular methods in rivers from Europe and South America, originating from the excretion of faeces by infected hosts [11]. Thus, long-term shedding of HEV from suids could facilitate its persistence in the host communities and environment, through an increase in the exposure and risk of transmission to other sympatric susceptible wild and domestic animals [12].

In southern Spain, HEV-3 is endemic, and exposure and infection rates have been obtained by serological and molecular methods in domestic pigs (seroprevalence range 16.5–60.8%), wild boar (seroprevalence range 5.2–57.6%), red deer (*Cervus elaphus*; seroprevalence around 10%) and equines (prevalence of 0.4–3.6%) [5,13,14,15,16,17,18,19,20]. Situations of a greater abundance and aggregation of wild boar are the best scenarios for HEV circulation, as they are associated with high seropositivity rates for HEV [5,6,21,22]. Additionally, higher seroprevalences of HEV were found in rural areas compared to urban areas [23,24], due to ecological and/or biological variations. However, several risk factors (mainly environmental, populational, meteorological or stochastic) might have been overlooked by these studies, due to their short-term approach.

Most of the ecological and epidemiological aspects of the sylvatic cycle of HEV remain unknown, and Doñana National Park (DNP; southwestern Spain) acts as a good field laboratory for the study of pathogens transmitted throughout indirect routes in the wildlife-livestock interface [25,26]. A previous survey conducted in 2015 reported the circulation of HEV-3 in wild boar from our study area and seroprevalence of 57.6% [27]. The activity (e.g., defecating) of this species around water points is likely to result in environmental contamination with HEV and the potential transmission to other susceptible hosts [28,29]. The importance of wild boar in the epidemiological cycle of HEV and its key role in viral transmission provides evidence of the need to conduct long-term studies.

Long-term approaches provide a broad temporal perspective to understand processes that potentially determine the host-pathogen dynamics, allowing us to discern the main drivers of the introduction, persistence and maintenance of the pathogens in the host communities [30]. This approach is particularly appropriate for a pathogen whose transmission depends greatly on environmental and host population factors, which may markedly change over years, due to host, density-dependent (e.g., density itself and/or aggregation) and stochastic factors (e.g., climate), as well as their complex interaction networks. However, there is a lack of long-term studies assessing the main drivers of HEV infection in wildlife. In this context, we conducted a long-term serosurvey of HEV in the wild boar from DNP during a 13-year period, with the specific aim of assessing the temporal trend of the seroprevalence of HEV and identifying the factors modulating it, namely, the individual, environmental, populational and stochastic factors.

## 2. Materials and Methods

### 2.1. Study Area

**Figure 1:**
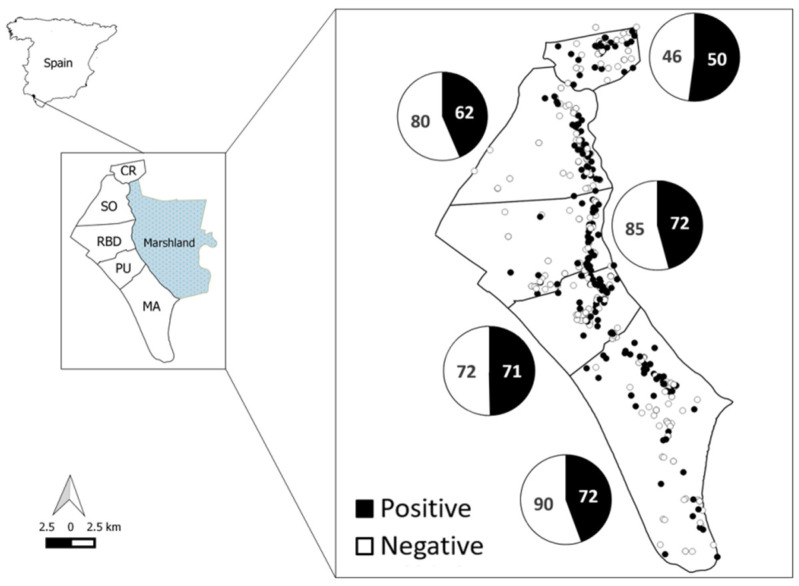
Map of the study area, Doñana National Park. The livestock management areas (Coto del Rey (CR), Sotos (SO), Doñana Biological Reserve (RBD), Puntal (PU) and Marismillas (MA)) are delimited. Sampling distribution and the number of animals positive and negative for HEV-antibodies observed in each livestock management area are also shown. Black and white symbols mean animals positive and negative for anti-HEV antibodies, respectively. The ecotone is the north to south line between the marshland (east, in blue) and the management areas (west).

This study was carried out in DNP (37°9′ N, 6°30′ W; Figure 1), a flat sandy area of 54,252 ha located on the Atlantic coast of southwest Spain. Three main biotopes are present in DNP: Scrubland, dunes and marsh. The scrubland and marshland are divided by a narrow strip of humid ecotone characterised by a high ecological richness. The climate of DNP is dry, subhumid Mediterranean, characterised by strong seasonality.

Free-ranging cattle and horse breeding are allowed, except in the northern area. Livestock is kept in the five livestock management areas of DNP, from north to south (Figure 1): Coto del Rey (CR), Sotos (SO), Doñana Biological Reserve (RBD), Puntal (PU) and Marismillas (MA). The wild ungulate community which inhabits DNP is comprised of wild boar, red deer and fallow deer (*Dama dama*). This abundant community of wild and domestic ungulates inhabits a place where the resources are limited during the dry season (late summer and autumn). This forces the aggregation of animals around water points [31] during the dry season and acorn mast in autumn [32]. A more detailed description of the management, environmental conditions and habitats in DNP has been provided by the authors of [33].

### 2.2. Animal Sampling

We selected the wild boar population from the entire ungulate community of DNP because; (i) it is the only species that has been widely recognised as a reservoir of HEV [5,6,34], and (ii) for its constant population increase and expansion [35], which increment the potential for HEV transmission in a wide range of environments, especially those where susceptible host species coexist. Taking advantage of the population control undertaken by park rangers, 700 wild boar were necropsied between 2005–2006 and 2017–2018 sampling seasons (sampling seasons typically occur between October and January). This health-monitoring programme was temporally interrupted during 2008–2009. Individuals were randomly selected, since culling activities did not address any specific objective about sex or age classes. This sampling belonged to the DNP health-monitoring programme and was performed according to: European and Spanish laws (RD 223/1988; [36], and EC Directive 86/609/EEC; [37]); current guidelines for the ethical use of animals in research [38]; the Animal Experiment Committee of Castilla-La Mancha University and the Spanish Ethics Committee (PR-2015-03-08). The geographical coordinates where each animal was initially sighted were registered using a portable GPS (Garmin Ltd., Olathe, KS, USA). Blood samples were collected into sterile plastic tubes without additives (Vacutainer^®^, Becton-Dickinson, NJ, USA) from the endocranial venous sinus, or alternatively from the heart or thoracic cavity [39].

### 2.3. Risk Factors

#### 2.3.1. Individual Factors

During the examination, the sex and age of the wild boar were determined, the latter by dentition eruption patterns [40]: Piglets (<6 months), juveniles (<6 months–2 years) and adults (>2 years).

#### 2.3.2. Environmental Factors

The habitat use by wild boar and its distribution in DNP is partly driven by environmental conditions [31]. In turn, the distribution of wild boar determines the spatiotemporal overlap of individuals around some resources, and therefore, the dynamics of pathogen transmission [32,33,41]. Thus, we selected a number of environmental variables as explanatory, some of them related to water sources and potential aggregation sites, to assess their potential effect on the seroprevalence of HEV (SH). Wild boar’s sampling site is integrated within its home range, and most of its life will be spent within an average radius of 1.2 km [32,42]. Thus, we calculated the straight-line distances (metres) from the exact location of each animal to the nearest marsh-shrub ecotone (DE), water point (DWAT), small human settlements (DHS), the marshland (DMARSH), stagnant water source (DSW) and the Guadalquivir river (DRIVER). The proportional cover of dense scrub, low-clear shrubland, herbaceous grassland, woodland, cover of bare land and watercourse vegetation for each animal were also calculated according to [33]. All the environmental information was collected from Andalusia Environmental Information [43].

#### 2.3.3. Populational Factors

To assess the potential effect of host population abundance on seroprevalence, we considered, apart from the wild boar, the ungulates inhabiting DNP, which may play a role in the epidemiology of HEV (red deer, fallow deer and cattle) [12,15,44,45,46]. Horses were not considered, due to the low prevalence reported for this species (0.4%; [13]). The relative abundance of red deer and wild boar per area of livestock management, expressed as Kilometric Abundance Indexes (KAI) [47], was monitored annually. KAI is used as a relative abundance index to inform in differences in wild boar abundance among areas and across years. We also calculated the population densities of fallow deer, and the cattle stocks (individuals/km^2^) for each livestock management area and sampling period. For details about sampling design and effort, see Barroso et al. [41]. The annual intra-specific seroprevalence of HEV was also calculated to assess its effect on the probability of wild boar testing positive for HEV.

#### 2.3.4. Stochastic Factors

The potential influence of climatic conditions concerning the availability of food resources and water to wild boar during the different seasons along the DNP is well-known [33], and the use of resources by animals increases their exposure to several pathogens [48]. HEV viral particles are very resistant to environmental conditions, facilitating its transmission [49], and dry years have been reported to be associated with increased viral contamination of water sources in different regions of Europe [11]. We, therefore, selected the rainfall and temperature as meteorological parameters to be included in our models to assess their potential effect on HEV epidemiology. The average rainfall and temperature per sampling period were calculated from the data obtained from the meteorology station located at RBD [50]. We included the rainfall and temperature of the previous sampling periods (calculated from September to August) in the models, since the animals sampled from August onwards had also been subjected to the meteorological conditions of the previous months.

### 2.4. Serological Analyses

Sera obtained by centrifugation (400× *g* for 5 min) from blood without additives were stored at −20 °C until assayed for antibodies against HEV. Anti-HEV immunoglobulin (Ig) G antibodies were tested using a commercial indirect enzymatic immunoassay (ELISA; PrioCHECK^®^ HEV Antibody porcine ELISA Kit. Thermo Fisher Scientific^TM^, Waltham, MA, USA), following the manufacturer’s instructions. This ELISA test has been used previously for wild boar [14,27,51], and is based on recombinant antigens of the open reading frame (ORF) ORF2 and ORF3 derived from genotypes 1 and 3. According to the information provided by the manufacturer, sensitivity and specificity are 91% and 94%, respectively.

### 2.5. Statistical Analysis

As a previous step, collinearity between individual, environmental, populational and stochastic variables was explored [52]. Given the high level of correlation obtained between land-cover variables, we performed a principal component analysis (PCA) and obtained two uncorrelated factors; closed habitats with dense scrub and woodland, and watercourse habitats (see Table S1).

Generalised linear mixed models (GzLMMs; binomial error distribution and logit link function) were fitted to assess the relationship between the serological response against HEV (negative/positive; as a response variable) and its potential risk factors. Firstly, the spatial differences in the HEV exposure between livestock management areas (CR, SO, RBD, PU and MA) were assessed in an exploratory GzLMM. In this GzLMM, the sex, age and livestock management area were included as fixed terms, and the sampling period and month as random terms. A second model was performed with the purpose of generalising the effect of the variables on the serological status against HEV regardless of the livestock management area and the sampling period. Thus, the final model included these variables as random-effect factors. The explanatory variables were: (i) Individual factors (sex and age class); (ii) environmental factors (DE, DWAT, DRIVER, DHS, DSW and closed cover and the coverage of watercourse habitats); (iii) populational factors, including the relative abundances of red deer and wild boar, densities of fallow deer and cattle, and intra-specific seroprevalence of HEV, by livestock management area and sampling season; and (iv) stochastic factors (previous sampling period’s rainfall and temperature). In order not to overparameterise the statistical models, and to build models based on the initial hypotheses regarding the influence of density-dependent and stochastics factors, as well as the role mediated by individual factors (sex and age), we included all two-way interactions between sex, age, rainfall, temperature and population abundances and densities (wild boar, red deer, fallow deer and cattle) [41]. A stepwise model selection process based on the corrected Akaike’s Information Criterion (cAIC) was used to select the most parsimonious model (Table S2) [53]. Once the final model was obtained, the assumptions of the binomial GzLMMs were checked [52]. A significant *p*-value was set at 0.05, and the predicted probabilities of serological response to HEV obtained from these models were used to represent the results. GzLMMs were fitted in the R library *lme4* 1.1-21 version (R software 4.0.2 version [54].

Finally, cross and self-correlations between seroprevalence and the relative abundance of wild boar were carried out to explore similarities in temporal patterns. The time lag (latency) at which the correlation was maximised was used to determine the latency between the two time-series explored. We used time shifts (lags) between −4 and 4 sampling periods. The absolute values of the cross and self-correlations were considered significant if they exceeded twice the estimated standard error values. Cross-correlations and self-correlations were run using IBM SPSS 19.0 software (IBM Corporation, Somers, NY, USA; [55]). The confidence intervals for seroprevalences were estimated by the standard error 95% confidence interval (S.E. 95%).

## 3. Results

### 3.1. General Results

**Figure 2:**
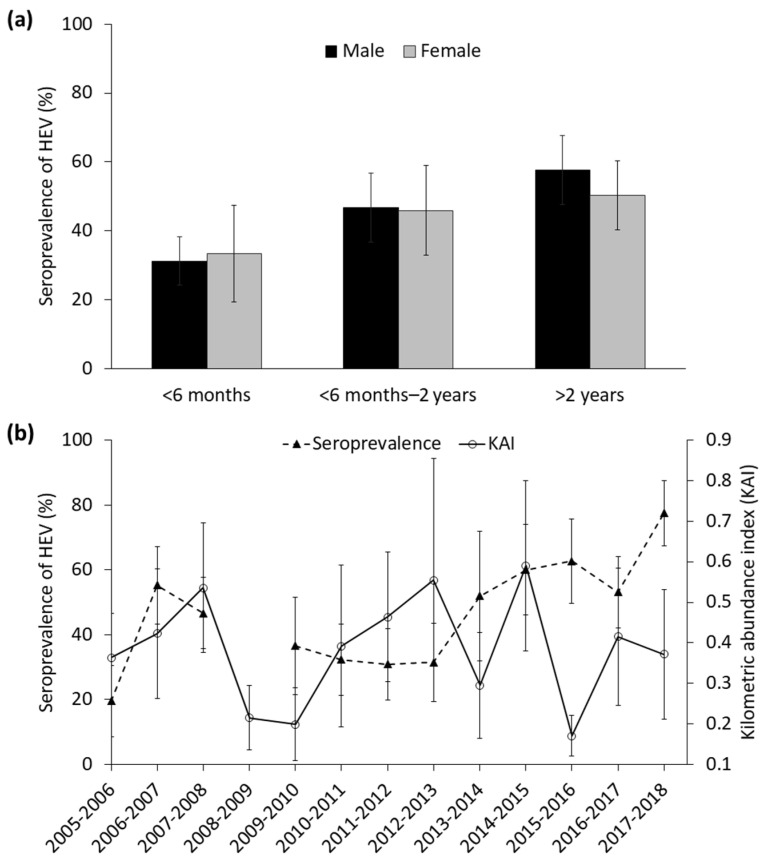
(**a**) Seroprevalence (% ± standard error (S.E.) 95%) of HEV depending on age class and sex. (**b**) Temporal trend of the seroprevalence of HEV (±S.E. 95%) and the average (±S.E.) relative abundance of wild boar (KAI).

A total of 327 out of 700 wild boar (SH = 46.7 ± 3.8%) presented anti-HEV antibodies in DNP during the study period (Figure 1). Table 1 displays SH observed in wild boar by livestock management area and sampling period. SH were 48.4 ± 0.1% and 44.7 ± 0.1% for males and females, respectively. The SH sex and age patterns are shown in Figure 2a. The temporal trend in the SH, as well as in the relative abundance of wild boar, are shown in Figure 2b. An apparent increase in SH in the 2012–2013 season after a steady decrease from 2006–2007 is noteworthy, reaching the maximum in the last sampling period (2017–2018). Spatially, the first explanatory model did not reveal statistical differences in the SH between livestock management areas (Figure 1; *F* = 0.68, df = 690, *p* = 0.62).

### 3.2. Factors Determining the Seroprevalence of HEV

**Figure 3:**
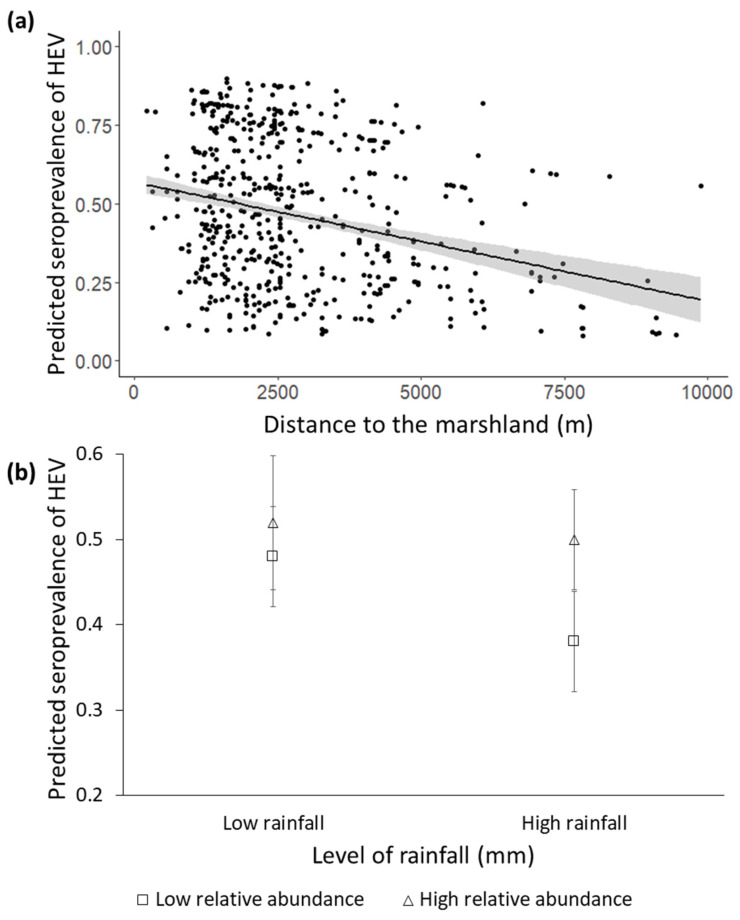
(**a**) Individual predicted probability to test positive for anti-HEV antibodies (±confidence interval (C.I.) 95%, represented by the shaded band) obtained from selected generalised linear mixed models (GzLMMs) depending on the distance to the marshland (metres). (**b**) Mean predicted probability to test positive for anti-HEV antibodies (±standard error (S.E.) 95%, represented by the error bars) obtained from selected GzLMMs depending on the interaction between annual rainfall (millimetres) and the relative abundance of wild boar (KAI). The following categories were established to display results: low rainfall (≤521.10 mm), high rainfall (>521.10 mm), low relative abundance of wild boar (≤0.69 KAI) and high relative abundance of wild boar (>0.69 KAI).

The results obtained from the best GzLMM of SH are shown in Table 2. However, we also consider three potential models obtained during the backward stepwise model selection procedure, since differences <2cAIC values were observed (Table S3). Age was statistically significant to explain SH, so that an increasing trend, regardless of sex, was evident (Figure 2a). Among the environmental factors, the distance to the marshland was the only parameter that remained in the model selected after the stepwise selection procedure. (Table S2), indicating that the closer to the marshland, the higher the SH (Figure 3a) was. Among populational factors, the relationship between the relative abundance of wild boar (KAI) and SH was conditioned by the rainfall (Figure 3b, see detailed explanation below). The intra-specific SH was significantly and positively associated with SH. Concerning the stochastic factors, the interaction between the previous sampling period’s rainfall and the KAI of wild boar was retained in the best model and was statistically significant. SH was the lowest when high rainfall and low relative abundance co-occurred. In high relative abundance conditions, SH was high regardless of the rainfall level (Figure 3b).

In the other three potential best models, the age, the distance to the marshland, and the intra-specific SH also provided statistically significant results associated with the risk of presenting antibodies to HEV, whereas no effect was observed for the relative abundance of wild boar, the sex, or the interactions between rainfall and sex with the relative abundance of wild boar (see Table S3).

### 3.3. Self-Correlations and Cross-Correlations

The annual values of SH showed significant positive self-correlations, with a lag of one year (R: 0.71 ± 0.24; Box-lung value: 10.25; *p* = 0.05).

## 4. Discussion

To the best of our knowledge, this is the first longitudinal long-term survey (2005–2018) of HEV exposure in wild boar. This approach allowed us to determine a temporal pattern conditioned by marked variations in the population control of wild boar (and subsequent changes in their relative abundances), as well as in the climatic conditions throughout the study period. We have contributed to broadening the understanding of the epidemiology of HEV in such a relevant wild host and opened new perspectives for further research regarding other hosts (including the human side) and the role of management and environmental features over the long-term.

### 4.1. General Patterns in the Seroprevalence of HEV

Overall, the SH value (46.72%) is higher than those obtained in the majority of European studies, mostly ranging from 4.9% to 34% [6,14,56,57], and concurs with some values detected in Spain in woodland (mainly hunting areas [12], and urban environments [58]). Occasionally, similar values have been previously reported in wild boar populations over European countries, such as Poland or Italy [22,59,60]. The high seroprevalence obtained, despite the absence of contact with domestic free-ranging pigs, suggests the capability of wild boar to maintain a constant exposure to HEV within this host community. In fact, the detection of HEV antibodies in a significant number of young wild boar (6–24 months old) for the whole study period (data not shown) suggested the recent and persistent exposure to this virus during these years, since it has been demonstrated that swine are infected at an early age after the loss of maternal antibodies [61]. The appearance of IgM antibodies in serum occurs first and is of relatively short shelf-life, followed by IgG that is more permanent in time (up to 22 weeks) [62]. Thus, IgG is more suitable for long-term seroprevalence studies [5,63,64], although its detection does not evidence early exposure to HEV. Further research is needed at the community level to determine the SH in sympatric cattle, horses, carnivores, lagomorphs and deer species, as well as the determinants of the persistence of HEV in such multi-host systems.

HEV is widely distributed over the study area, which contrasts with the clear spatial patterns shown by other pathogens at a relatively small scale in the wild boar in DNP [26,41,65]. This may indicate that factors not related to specific local conditions of the environment mostly determined heterogeneity in HEV exposure and/or SH in wild boar over the long-term. However, there were important interannual differences in the SH (ranging from 19.6 to 77.5%), characterised by steady increases or decreases, whose potential determinants are discussed below. There was a clear inflexion point of SH characterised by a continuous steady increase from 2012–2013 onwards. This change in the trend is coincident with the recovery of the wild boar population following a significant population control programme in the Park during 2004–2005 and 2005–2006 (Figure 2b; a total of 1000 individuals were removed from the population). The self-correlation of the temporal pattern (1 year-lag) suggests that patterns observed from a perspective of at least two years are independent of previous SH, which concurs with the rapid population turnover that characterises wild boar.

### 4.2. Factors Determining Seroprevalence of HEV

We evidenced a significant age-increasing pattern in SH, regardless of sex (Table 2, Figure 2a). This concurs with some studies [27,57,60]. However, most studies on HEV in wild boar have not reported significant age-related differences [5,12,18,56]. The likelihood of exposure to the virus throughout life, together with the high persistence of antibodies against HEV in wild boar, may underly this pattern [14].

The proximity of the wild boar sampled to the marshland positively associated with increased SH. During the last 10 years, our research group has captured and tagged more than 50 wild boar with telemetry collars in DNP [32,33,66]. Telemetry data allow us to explain and discuss the habitat use by wild boar in the study area. During the dry season, the soil of the marshland is moisture-retentive [67], and is significantly used by wild boar, wallowing and feeding on *Scirpus maritimum* roots, earthworms, small crustaceans or mammals, predating on aquatic bird nests, or taking mud-baths [68]. Furthermore, the level of water contamination increases during the dry season, due to an increase in the concentration of pollutants, as has been reported in human studies [69]. The conditions of the marsh during this period may also allow for the increased survival of the virus in the environment and the subsequent high exposure of the wild boar to it.

High relative abundances of wild boar entailed increased SH risk. Previous studies have considered wild boar management practices as a proxy of density to assess its effect on SH [5,12]. In these studies, significantly higher SH has been found in fenced hunting estates than in open areas from south-central Spain, probably mediated by the increased aggregation (e.g., artificial feeding and watering at specific points) and the higher densities of animals [5,6,21]. Likewise, there is a negative impact associated with high densities concerning the nutrition of wild boar, as well as increased mortality rates, due to undernutrition and diseases, such as tuberculosis [70]; there may also be an increase in scavenging and risks for HEV transmission. Moreover, Larska et al. [22] reported a positive association between the density of wild boar and the SH in a study undertaken concerning several wildlife species in Poland.

The lowest SH was observed when high rainfall and a low relative abundance of wild boar co-occurred, whereas in high relative abundance situations, SH was high regardless of the rainfall level (Figure 3b). Discrepancies have been observed in the relationship between the prevalence of HEV and meteorological conditions, concluding that both rainfall and temperature did not appear to be drivers of the prevalence of HEV in wild boar [71], while another study reported a limited survival of HEV particles in the soil over 37 °C [72]. To our knowledge, there are no previous studies evaluating the effects mediated by rainfall on the risk of HEV in wild boar, which is also expected to vary depending on the local conditions and climate (Mediterranean, in our case). In our study, the temperature did not result relevant to explain the SH in the final model (see Table 2). However, the risk of direct and/or indirect HEV exposure and transmission among wild boar increases during dry years, since aggregation at the scarce watering sites available during the dry season rises as well [41,48,73]. Under these circumstances, not only do direct interactions among individuals occur more often, but there is also greater environmental contamination and persistence, since wild boar wallow in, brush against, defecate and urinate in their surroundings [33,74,75]. This may also be potentiated by cannibalism or scavenging, since wild boar carcasses become more available during periods of drought in Mediterranean regions [70,76]. Thus, the concomitance of low rainfall and high densities in DNP possibly involves a greater aggregation at watering sites and the subsequent risk of direct and indirect transmission of HEV. On the contrary, rainy years together with the low relative abundance of wild boar may imply an increase in water sources, lower host aggregation and the subsequent dilution of viral particles in the environment, leading to lower SH [11]. However, in rainy years when a high relative abundance of wild boar occurs, this dilution effect of HEV particles caused by the rain might be not enough to counteract the high level of environmental contamination, as well as the increased direct and indirect interactions among individuals that are derived from their high abundance.

## 5. Conclusions

The effect of the relative abundance in the SH of wild boar in this study reflects the human management the population is subjected to, which is the main determinant of the wild boar population size in DNP, where predators do not exist. This factor should be considered in conjunction with others, some operating stochastically on a long-term scale (e.g., meteorological conditions), as they may interact and mask the concomitant action of different drivers.

The abundance of wild boar is still increasing, and this applies to most of the distribution range of Eurasian wild boar (which is also expanding) and feral pigs worldwide. In this study, the increase of SH observed after a major population culling programme (2004–2005 and 2005–2006) evidences the importance of sustainably controlling populations of wild boar, which are a relevant source of pathogens shared with livestock, humans and other wildlife [28,77]. Wild boar population control remains a challenge at the international level [78], and the current socio-economic context is not favourable. An increase in disease-related conflicts associated with this species is expected, even in peri-urban and urban habitats [58].

Wild disease surveillance, including integrated population monitoring, will be essential for disease risk evaluation and subsequent decision making. In the case of HEV, multi-host systems, including other wildlife species, such as livestock; human and environmental sources, should also be considered. Cross-species transmission of HEV from animal reservoirs is the major route of infection in humans, it has become one of the most successful zoonotic viral diseases in both developed and developing countries [79], and our results indicate that this problem may increase. Therefore, HEV and wildlife reservoirs should not be neglected as potential public health risks for humans in the future.

## Figures and Tables

**Table 1:** Seropositivity for HEV (%) obtained by ELISA and sample size (N) by sampling period and livestock management area (Coto del Rey (CR), Sotos (SO), Doñana Biological Reserve (RBD), Puntal (PU) and Marismillas (MA)).

Season	CR	SO	RBD	PU	MA	Total
	Seroprevalence (N)	Seroprevalence (N)	Seroprevalence (N)	Seroprevalence (%)	Seroprevalence (%)	Seroprevalence (%)
2005–2006	-	16.7 (6)	25 (12)	12.5 (8)	20 (25)	19.6 (51)
2006–2007	71.4 (14)	57.1 (14)	35.3 (17)	66.7 (18)	25 (4)	55.2 (67)
2007–2008	-	19.2 (26)	42.9 (7)	69.2 (13)	56.3 (32)	46.7 (75)
2008–2009	-	-	-	-	-	-
2009–2010	100 (2)	50 (4)	41.2 (17)	-	2.2 (18)	36.6 (41)
2010–2011	29.4 (17)	40 (10)	35 (20)	25 (12)	33.3 (9)	32.4 (68)
2011–2012	25 (8)	31.6 (19)	26.3 (19)	54.5 (11)	18.2 (11)	30.9 (68)
2012–2013	0 (9)	0 (6)	38.5 (13)	42.9 (7)	47.4 819)	31.5 (54)
2013–2014	-	-	57.1 (7)	50 (18)	-	52.0 (25)
2014–2015	100 (2)	81.3 (16)	75 (8)	41.2 (17)	28.6 (7)	60.0 (50)
2015–2016	73.3 (15)	41.7 (12)	80 (10)	33.3 (6)	75 (8)	62.7 (51)
2016–2017	46.7 (15)	46.7 (15)	78.6 (14)	35 (20)	66.7 (15)	53.2 (79)
2017–2018	78.6 (14)	78.6 (14)	53.8 (13)	92.3 (13)	85.7 (14)	77.5 (71)
Total 2005–2018	52.1 (96)	43.7 (142)	54.1 (157)	49.7 (143)	44.4 (162)	46.7 (700)

**Table 2:** Parameters from the best GzLMM for the serological status against HEV in wild boar related to age class, relative abundance (KAI), straight-line distance to the marshland (DMARSH), the rainfall of the previous sampling period (rainfall), the intra-specific seroprevalence of HEV and the interaction between rainfall and KAI.

Variables	F df (x,y)	Estimate ± SD ^2^	*p*
Age class ^1^	5.30 (2, 690)	1–2 years: 0.46 ± 0.25 >2 years: 0.76 ± 0.21	<0.01
Distance to marsh	3.09 (1, 690)	−0.0001 ± 0.0006	0.03
Abundance of wild boar (KAI)	4.07 (1, 690)	−0.85 ± 0.72	0.22
Rainfall	0.01 (1, 690)	−0.002 ± 0.001	0.05
Rainfall*abundance of wild boar	3.74 (1, 690)	0.002 ± 0.001	0.05
Intra-specific seroprevalence	102.59 (1, 690)	0.04 ± 0.005	<0.01

The model was fitted using the sampling period and livestock management area as random effect factors. ^1^ Parameter estimates for the age class were calculated using <6 months old animal as the reference. ^2^ Standard deviation. The conditional R^2^ obtained from this model was 0.267.

## Supplementary Materials

The following are available online at https://www.mdpi.com/article/10.3390/ani11061805/s1, Table S1: Result of the Principal Component Analysis (PCA) and the two uncorrelated factors obtained, Table S2: Summary of the stepwise model selection procedure, based on the corrected Akaike’s information criterion (cAIC), used to explain the serological status against HEV. The full model comprises the age class, sex, straight-line distance to the nearest: Ecotone (DE), water point (DWAT), small human settlements (DHS), the marshland (DMARSH), stagnant water source (DSW) and the Guadalquivir river (DRIVER), rainfall (rainfall) and temperature (temperature) of the previous sampling period, cover of closed habitats (closed_ha), coverage of watercourse habitats (water_ha), annual density of fallow deer (FD_den) and cattle (cattle_den), relative abundance of red deer (RD_KAI) and wild boar (WB_KAI), intra-specific seroprevalence of HEV (seroprev_intrasp) and some interaction among them (*). In bold type is shown the variable which was removed between steps, Table S3: Parameters from the other potential best GzLMMs for the serological status against HEV in wild boar related to age class, relative abundance (KAI), straight-line distance to the marshland (DMARSH), the rainfall of the previous sampling period (rainfall), the interaction between rainfall and KAI, the intra-specific seroprevalence of HEV, the cover of closed habitats (closed_ha) and sex.
