# Supplementary material for: Long-Term Determinants of the Seroprevalence of the Hepatitis E Virus in Wild Boar (Sus scrofa)
Source: Animals (Basel). 2021 Jun 17;11(6):1805. doi: 10.3390/ani11061805 (PMC8235029; doi:10.3390/ani11061805)
Supplement: Supplementary file 1 [file animals-11-01805-s001.zip › animals-1222337-supplementary.pdf]

Table S1. Result of the Principal Component Analysis (PCA) and the two uncorrelated factors obtained.

Variable	PC1- closed habitats	PC2-watercourse habitats
**Herbaceous grassland**	-0.856	-0.507
**Bare land**	0.259	-0.098
**Watercourse vegetation**	-0.096	0.991
**Woodland, dense and low-clear shrubland**	0.866	-0.362
**Standard deviation**	1.56	1.38
**Explained variance**	38.96	34.48
**Cumulative variance**	38.96	73.44

Table S2. Summary of the stepwise model selection procedure, based on the corrected Akaike's information criterion (cAIC), used to explain the serological status against HEV. Full model comprises the age class, sex, straight-line distance to the nearest: ecotone (DE), water point (DWAT), small human settlements (DHS), the marshland (DMARSH), stagnant water source (DSW), and the Guadalquivir river (DRIVER), rainfall (rainfall) and temperature (temperature) of the previous sampling period, cover of closed habitats (closed_ha), coverage of watercourse habitats (water_ha), annual density of fallow deer (FD_den) and cattle (cattle_den), relative abundance of red deer (RD_KAI) and wild boar (WB_KAI), intra-specific seroprevalence of HEV (seroprev_intrasp), and some interaction among them (*). In bold type is shown the variable which was remove between steps.

Model	cAIC
**Full model:** age + sex + DE + DWAT + DHS + DMARSH + DSW + DRIVER + rainfall + temperature + closed_ha + water_ha + FD_den + cattle_den + RD_KAI + WB_KAI + seroprev_intrasp + sex*age + **age*WB_KAI** + sex*WB_KAI + rainfall*WB_KAI + rainfall*sex + rainfall*age + rainfall*temperature [M1]	856.49
age + sex + DE + DWAT + DHS + DMARSH + DSW + DRIVER + rainfall + temperature + closed_ha + water_ha + FD_den + cattle_den + RD_KAI + WB_KAI + seroprev_intrasp + sex*age + sex*WB_KAI + rainfall*WB_KAI + rainfall*sex + **rainfall*age** + rainfall*temperature [M2]	853.25
age + sex + DE + DWAT + DHS + DMARSH + DSW + DRIVER + rainfall + temperature + closed_ha + water_ha + FD_den + cattle_den + RD_KAI + WB_KAI + seroprev_intrasp + **sex*age** + sex*WB_KAI + rainfall*WB_KAI + rainfall*sex + rainfall*temperature [M3]	850.41
age + sex + DE + DWAT + DHS + DMARSH + DSW + DRIVER + rainfall + temperature + closed_ha + water_ha + FD_den + cattle_den + **RD_KAI** + WB_KAI + seroprev_intrasp + sex*WB_KAI + rainfall*WB_KAI + rainfall*sex + rainfall*temperature [M4]	847.89
age + sex + DE + DWAT + DHS + DMARSH + DSW + DRIVER + rainfall + temperature + closed_ha + water_ha + **FD_den** + cattle_den + WB_KAI + seroprev_intrasp + sex*WB_KAI + rainfall*WB_KAI + rainfall*sex + rainfall*temperature [M5]	845.89
age + sex + DE + DWAT + DHS + DMARSH + DSW + DRIVER + rainfall + temperature + closed_ha + water_ha + cattle_den + WB_KAI + seroprev_intrasp + sex*WB_KAI + rainfall*WB_KAI + **rainfall*sex** + rainfall*temperature [M6]	843.91

age + sex + DE + DWAT + DHS + DMARSH + DSW + DRIVER + rainfall + temperature + closed_ha + water_ha + **cattle_den** + WB_KAI + seroprev_intrasp + sex*WB_KAI + rainfall*WB_KAI + rainfall*temperature [M7]	841.95
age + sex + DE + DWAT + DHS + DMARSH + DSW + **DRIVER** + rainfall + temperature + closed_ha + water_ha + WB_KAI + seroprev_intrasp + sex*WB_KAI + rainfall*WB_KAI + rainfall*temperature [M8]	839.99
age + sex + DE + DWAT + **DHS** + DMARSH + DSW + rainfall + temperature + closed_ha + water_ha + WB_KAI + seroprev_intrasp + sex*WB_KAI + rainfall*WB_KAI + rainfall*temperature [M9]	838.05
age + sex + DE + **DWAT** + DMARSH + DSW + rainfall + temperature + closed_ha + water_ha + WB_KAI + seroprev_intrasp + sex*WB_KAI + rainfall*WB_KAI + rainfall*temperature [M10]	836.16
age + sex + DE + DMARSH + **DSW** + rainfall + temperature + closed_ha + water_ha + WB_KAI + seroprev_intrasp + sex*WB_KAI + rainfall*WB_KAI + rainfall*temperature [M11]	834.40
age + sex + DE + DMARSH + rainfall + temperature + closed_ha + water_ha + WB_KAI + seroprev_intrasp + sex*WB_KAI + rainfall*WB_KAI + **rainfall*temperature** [M12]	832.53
age + sex + DE + DMARSH + rainfall + temperature + closed_ha + **water_ha** + WB_KAI + seroprev_intrasp + sex*WB_KAI + rainfall*WB_KAI [M13]	830.74
age + sex + **DE** + DMARSH + rainfall + temperature + closed_ha + WB_KAI + seroprev_intrasp + sex*WB_KAI + rainfall*WB_KAI [M14]	828.93
age + sex + DMARSH + rainfall + **temperature** + closed_ha + WB_KAI + seroprev_intrasp + sex*WB_KAI + rainfall*WB_KAI [M15]	827.22
age + sex + DMARSH + rainfall + closed_ha + WB_KAI + seroprev_intrasp + **sex*WB_KAI** + rainfall*WB_KAI [M16]	826.07
age + **sex** + DMARSH + rainfall + closed_ha + WB_KAI + seroprev_intrasp + rainfall*WB_KAI [M17]	826.06
age + DMARSH + rainfall + **closed_ha** + WB_KAI + seroprev_intrasp + rainfall*WB_KAI [M18]	824.59
age + DMARSH + rainfall + WB_KAI + seroprev_intrasp + rainfall*WB_KAI [M19]	824.50

Table S3. Parameters from the other potential best GzLMMs for the serological status against HEV in wild boar related to age class, relative abundance (KAI), straight-line distance to the marshland (DMARSH), the rainfall of the previous sampling period (rainfall), the interaction between rainfall and KAI, the intra-specific seroprevalence of HEV, the cover of closed habitats (closed_ha), and sex.

**Variables**	**Model 18 cAIC=824.59 R^2=0.270**			**Model 17 cAIC=826.06 R^2=0.271**			**Model 16 cAIC=826.07 R^2=0.274**		
	F df (x,y)	Estimate ±SD[3]	*p*	F df (x,y)	Estimate ±SD	*p*	F df (x,y)	Estimate ±SD	*p*
**Age class[1]**	5.31 (2, 689)	1-2 years:0.45±0.25 > 2 years: 0.77±0.21	**<0.01**	5.23 (2, 688)	1-2 years:0.44±0.25 > 2 years: 0.78±0.21	**<0.01**	5.23 (2, 687)	1-2 years:0.43±0.25 > 2 years: 0.78±0.21	**<0.01**
**DMARSH**	5.38 (1, 689)	-0.0002±0.00006	**0.01**	5.41 (1, 688)	-0.0002±0.0001	**0.01**	5.39 (1, 687)	-0.0002±0.0001	**<0.01**
**Abundance of wild boar (KAI)**	3.91 (1, 689)	-0.75±0.73	0.31	4.04 (1, 688)	-0.52±0.76	0.32	3.97 (1, 687)	-0.52±0.76	0.49
**Rainfall**	0.02 (1, 689)	-0.002±0.001	0.06	0.003 (1, 688)	-0.001±0.001	0.07	0.004 (1, 687)	-0.001±0.001	0.06
**Rainfall* abundance of wild boar**	2.99 (1, 689)	0.002±0.001	0.08	2.94 (1, 688)	0.002±0.001	0.08	2.86 (1, 687)	0.002±0.001	0.07
**Intra-specific seroprevalence**	102.47 (1, 689)	0.04±0.01	**<0.01**	101.91 (1, 688)	0.04±0.01	**<0.01**	101.04 (1, 687)	0.04±0.01	**<0.01**
**Closed_ha**	0.05 (1, 689)	0.14±0.09	0.15	0.66 (1, 688)	0.14±0.09	0.14	0.63 (1, 687)	0.14±0.09	0.14
**Sex[2]**				0.29 (1, 688)	Female: -0.12±0.17	0.51	0.30 (1, 687)	Female: -0.28±0.33	0.40
**Sex[2]*abundance of wild boar**							1.98 (1, 687)	Female: -0.56±0.40	0.16

The model was fitted using the sampling period and livestock management area as random effect factors. Parameter estimates for the [1]age class were calculated using <6 months old animal as the reference and the male level in the variable sex[2]. [3]Standard deviation.
